# Linking Early Life Hypothalamic–Pituitary–Adrenal Axis Functioning, Brain Asymmetries, and Personality Traits in Dyslexia: An Informative Case Study

**DOI:** 10.3389/fnhum.2019.00327

**Published:** 2019-10-01

**Authors:** Victoria Zakopoulou, Angeliki-Maria Vlaikou, Marousa Darsinou, Zoe Papadopoulou, Daniela Theodoridou, Kyriaki Papageorgiou, George A. Alexiou, Haralambos Bougias, Vassiliki Siafaka, Pierluigi Zoccolotti, George P. Chroussos, Maria Syrrou, Theologos M. Michaelidis

**Affiliations:** ^1^Department of Speech and Language Therapy, School of Health Sciences, University of Ioannina, Ioannina, Greece; ^2^Laboratory of Biology, Faculty of Medicine, School of Health Sciences, University of Ioannina, Ioannina, Greece; ^3^Department of Biomedical Research, Foundation for Research and Technology-Hellas, Institute of Molecular Biology and Biotechnology, Ioannina, Greece; ^4^Department of Biological Applications and Technologies, School of Health Sciences, University of Ioannina, Ioannina, Greece; ^5^Faculty of Medicine, School of Health Sciences, University of Ioannina, Ioannina, Greece; ^6^Neuropsychology Unit, Department of Psychology, IRCCS (National Institute for Research and Treatment) Fondazione Santa Lucia, Sapienza University of Rome, Rome, Italy; ^7^First Department of Pediatrics, National and Kapodistrian University of Athens Medical School, “Aghia Sophia” Children’s Hospital, Athens, Greece

**Keywords:** dyslexia, brain asymmetries, stress, hypothalamic–pituitary–adrenal (HPA) axis, neuroplasticity genes, BDNF, MR

## Abstract

Developmental dyslexia (DD) is a multi-system disorder, combining influences of susceptibility genes and environmental factors. The causative interaction between specific genetic factors, brain regions, and personality/mental disorders, as well as specific learning disabilities, has been thoroughly investigated with regard to the approach of developing a multifaceted diagnostic procedure with an intervention strategy potential. In an attempt to add new translational evidence to the interconnection of the above factors in the occurrence of DD, we performed a combinatorial analysis of brain asymmetries, personality traits, cognitive and learning skills, and expression profiles of selected genes in an adult, early diagnosed with DD, and in his son of typical development. We focused on the expression of genes, based on the assumption that the regulation of transcription may be affected by genetic and epigenetic factors. The results highlighted a potential chain link between neuroplasticity-related as well as stress-related genes, such as BDNF, Sox4, mineralocorticoid receptor (MR), and GILZ, leftward asymmetries in the amygdala and selective cerebellum lobules, and tendencies for personality disorders and dyslexia. This correlation may reflect the presence of a specific neuro-epigenetic component of DD, ensuing from the continuous, multifaceted difficulties in the acquisition of cognitive and learning skills, which in turn may act as a fostering mechanism for the onset of long-term disorders. This is in line with recent findings demonstrating a dysfunction in processes supported by rapid neural adaptation in children and adults with dyslexia. Accordingly, the co-evaluation of all the above parameters may indicate a stress-related dyslexia endophenotype that should be carefully considered for a more integrated diagnosis and effective intervention.

## Introduction

Developmental dyslexia (DD) is a multi-system neurodevelopmental disorder that affects the ability of individuals to acquire specific learning skills, such as reading, writing, and spelling, despite having normal intelligence, perception instruction, sensory abilities, motivation, and educational opportunities (Spironelli et al., 2008; Peterson and Pennington, 2012; Langer et al., 2017). In the DSM-5™ handbook of differential diagnosis, the term DD is incorporated in an extensive designation, i.e., that of Specific Learning Disorder (SLD; American Psychiatric Association, 2013). In this article, we opt for the term DD since both the diagnostic elements and the bibliographic data are documented according to this terminology.

## Background

### Personality and DD: Psycho-emotional and Behavioral Outcomes

As originally advocated by Pennington (2006), a new conceptualization of investigating the complex substrate of DD proposes that the association between genetic, neurological, cognitive, psycho-emotional, and behavioral factors as well as their potential involvement in the onset of the symptoms and their continuity during development could better clarify the underlying components of the disease (Zakopoulou et al., 2014; Perrachione et al., 2016; Zoccolotti et al., 2016). DD often shows co-occurrence [Kaplan et al., 2006; also named co-existence (Gillberg, 2010) or comorbidity (Ramus et al., 2013; Ashraf and Najam, 2017)] with other disturbances, and shares common genetic factors with other mental disorders (Grigorenko, 2001; Pennington, 2006). In the context of dyslexia research, the emerging relationship between continuous learning difficulties and personality traits (personality characteristics affecting a person’s behavior, thoughts, and feelings across situations, such as openness to experience, agreeableness, and neuroticism) is of increasing interest (Mason and Mason, 2005; Swanson and Hsieh, 2009; Tsitsas, 2017). Several independent studies support the notion that the frustration and difficulties caused by learning problems *per se*, both at home and in school, create continuous fear of failure or actual failure, sadness, inadequacy, reduced happiness and self-esteem, anxiety, emotional vulnerability, embarrassment, defensive behaviors, as well as withdrawal (Huc-Chabrolle et al., 2010; Zakopoulou et al., 2013; Bonifacci et al., 2016; Mammarella et al., 2016). Similarly, high levels of tension, anxiety, and depression in students and adolescents with learning disorders (LD) have been reported (Wilson et al., 2009; Panicker and Chelliah, 2016), while Lufi and Awwad (2013) have documented a high probability of test anxiety for adults with LD. Moreover, a significantly lower level of psychosocial health was reported recently for children with LD (Matteucci et al., 2019), and further, in addition to anxiety, such children may have attentional biases specific to reading (Haft et al., 2019), a situation that may, in turn, perpetuate anxiety (Bar-Haim et al., 2007), emphasizing the importance of individualized interventions, considering the psycho- and socio-emotional difficulties in this population.

### Stress-HPA Axis, Neuroplasticity, and Epigenetic Reprogramming

Epigenetic mechanisms play a crucial role in the adaptive regulation of gene expression during postnatal life (Rutten and Mill, 2009; Szulwach et al., 2011). Numerous studies investigating the multidisciplinary phenotype of DD support the notion that early or longitudinal experiences of stress (prenatal and antenatal maternal stress, adult social stress) interact with neuroendocrine effectiveness in stressful social behavior and cognitive ability (Gudsnuk and Champagne, 2012; Li et al., 2013; Hostinar et al., 2015; D’Souza et al., 2016). Stress is capable of altering neurotransmission and synaptic plasticity in hypothalamic–pituitary–adrenal (HPA) axis-associated brain regions such as those of the prefrontal cortex (PFC), hippocampus, and amygdala (Gardner et al., 2009; van Bodegom et al., 2017). These regions are all targets of stress hormones known to be involved in dyslexia (Van den Bergh, 2011; Vogel and Schwabe, 2016). Stress exposure activates the HPA axis, resulting in elevation of blood glucocorticoid (GC) levels. The HPA axis is essential for successful adaptation to stress, and its dysfunction, combined with chronic stress exposure, especially early in life, may act as a triggering mechanism that could lead to the development of psychopathology (McEwen and Gianaros, 2011; Buschdorf and Meaney, 2015). GCs bind to two receptor types in the brain, mineralocorticoid receptor (MR) and glucocorticoid receptor (GR), both of which reside in the cytosol and upon GC-binding translocate to the nucleus where they regulate transcription by binding to GC-responsive elements of target genes (Pearce and Yamamoto, 1993). MR and GR mediate the initiation and termination of the HPA axis stress response and modulate acquisition, consolidation, storage, and retrieval of stressful experiences (Sapolsky et al., 2000; Kino and Chrousos, 2004; Montaron et al., 2006; Reul et al., 2015). *GILZ* has been used as an HPA axis activity measure. Its expression in peripheral blood decreases in chronic stress and social defeat and is correlated with smaller hippocampal volumes (Frodl et al., 2012). BDNF (brain-derived neurotrophic factor) is a key neurotrophic factor implicated in learning and memory, but also in neural plasticity in the amygdala (Rattiner et al., 2005; Cowansage et al., 2010). Mutations in its receptor have been shown to modulate acquisition and consolidation of fear learning (Musumeci et al., 2009). Adult neurogenesis, the birth of new neurons in the adult brain, is considered highly sensitive to environmental stressors (Karten et al., 2005) such as those specifically encountered in learning disabilities/dyslexia. The SoxC proteins play an important role in the genetic network controlling neuronal differentiation in adult neurogenesis, while Sox4 regulates the establishment of neuronal properties and specification of cell fate (Mu et al., 2012).

### The Neural-Systems Framework-Brain Asymmetries

Previous neuroimaging studies using functional MRI (fMRI; Shaywitz et al., 2002, 2004) and positron emission tomography (PET; Paulesu et al., 2014) when investigating both children and adults with dyslexia compared to control subjects reported hypo-activity in the left inferior parietal lobe and/or hyperactivity in the left inferior frontal gyrus during phonological awareness tasks such as rhyming judgments. Moreover, original voxel-based morphometry (VBM) studies unequivocally established the involvement of the posterior cerebellum in dyslexia, namely, lobules V, VI, and VII in reading difficulties (Carreiras et al., 2007), Crus I and II in semantic processing, and VIIB in cognitive tasks (Ruz et al., 2005).

Atypical abnormalities of the activity in the left temporal–occipital brain area may play a major role in the recognition of words and the accomplishment of phonological tasks (Kita et al., 2013). These atypical brain activities might underpin impaired phonological awareness in people with dyslexia. However, results from recent studies suggest that the differences found in multiple regions of the dyslexic brains indicate that cerebellar function is not the primary cause of dyslexia, but is rather a fundamental neurodevelopmental abnormality (Perrachione et al., 2016). Various investigations on animals and humans (Kim et al., 2011) testing the performance in cognitive or emotional tasks confirmed a structural and widespread reciprocal connectivity (Freese and Amaral, 2009) between the amygdala, hypothalamus, and ventromedial prefrontal cortex (vmPFC) in the regulation of emotions and social behavior (Arnsten, 2009; Schumann et al., 2009). During threat or uncertainty, the amygdala is activated under tonic inhibitory control from the PFC, having as a result the PFC to be hypoactive (Hänsel and von Känel, 2008; Thayer and Lane, 2009). Structural and functional brain asymmetries have been found in a number of prefrontal areas, mostly in adolescents and males, suggesting that these asymmetries may render them more vulnerable to certain disorders such as autism and dyslexia (Huster et al., 2007; Whittle et al., 2008).

### Aim of the Present Study

In this study, we examined whether early stress and stressful learning experiences may constitute underlying predisposing components in the occurrence of DD (Eckert, 2004), in the case of an adult man (GA) diagnosed early with DD, with reported difficulties in manipulating strong emotional situations, and impulsive behavior, even though he presented an endearing personality.

To confirm that GA fulfilled the diagnostic criteria for DD, the DAST test (Fawcett and Nicholson, 1998) was implemented. An “at-risk” profile for dyslexia was highlighted, indicating weaknesses in specific areas related to learning (phonological processing; processing speed; language processing; visual-spatial processing). Moreover, the psychological test MMPI, assessing personality traits and psychopathology (Hathaway and McKinley, 1951), was conducted to check for any possible co-existing disorders. According to this analysis, the psychological profile of the patient showed the characteristics of fearful people with poor self-image and intense anxiety, who find it hard to relax and overcome their fears.

The psychological assessment was complemented with MRI and three-dimensional surface rendering techniques. Taking into account previous evidence showing a reduction of gray matter in specific cerebellar regions associated with DD or other types of dyslexia, such as inferior frontal gyrus, precentral gyrus, medial occipital gyri, frontal and occipital lobes, insula and basal ganglia (Brambati et al., 2004; Zadina et al., 2006), a VBM analysis was performed to investigate potential global volumetric changes in relevant brain regions. This analysis revealed anomalous leftward anatomical asymmetries, consistent with other studies in dyslexic brains (Stoodley and Stein, 2011).

Stressful conditions and experiences have been associated with alterations of HPA axis- and neuroplasticity-related genes, such as the nuclear receptors MR and GR, and the neurotrophin BDNF *via* epigenetic mechanisms (de Kloet et al., 2005; Unternaehrer et al., 2012). In our analysis, we also included the genes for *Ube3A* (Ubiquitin-protein ligase E3A), a transcriptional coactivator of steroid hormone receptors associated with neurodevelopmental syndromes and psychological conditions (LaSalle et al., 2015), and *GILZ* (GC-induced leucine-zipper), a GR/MR-responsive gene that contributes significantly to neural activation and transmitter release and has been linked to HPA axis dysfunction and psychosocial stress (Srinivasan and Lahiri, 2017). Finally, we also monitored genes that are important for neuroplasticity and neurogenesis such as the aforementioned *BDNF* and *Sox4* (Bergsland et al., 2006; Mu et al., 2012). We compared the expression levels of the above genes using whole blood RNA from the patient and his 25-year-old son, a healthy individual, with normal reading ability. This test was repeated 1 year later. As detailed below, the results revealed significant and consistent differences in the mRNA levels of these genes between the two samples that could be related to potential pathological mechanisms underlying this disorder and may encourage the use of such genes as potential peripheral blood-based biomarkers.

### GA Case History

GA is a 56-year-old man, right-handed, diagnosed early with DD. According to his medical history and physical examination, GA was physically healthy, without any history of neurologic, psychiatric, or otherwise chronic diseases. The developmental history provided information about GA’s significant, persistent difficulties in reading, writing, and spelling since school age, class repetition, and failure in the national, university entry, examinations. Despite his severe learning disabilities, GA managed to complete his studies and obtain a PhD, and today he is a well-known scientist. However, as became obvious from the clinical interview, his high academic performance was achieved as a result of persistent and intense effort.

GA was referred to the neurological clinic because of visual abnormalities without any coexistent pathology. The brain imaging data and his early life history suggested a complex profile, demanding a more detailed investigation.

An informed consent document was signed by GA and his son, in accordance with the principles of the Declaration of Helsinki.

## Materials and Methods

See Supplementary File S1 for Materials and Methods.

## Results and Discussion

According to the DAST analysis, GA’s profile indicates very severe difficulties (—) with Rapid Naming and Phonemic Segmentation together with serious problems (—) in One Minute Reading, One Minute Spelling, and Nonverbal Reasoning. Noticeable problems (-) were observed in Postural Stability, Backwards Span, Nonsense Passage, and Verbal Fluency. Relatively good performance (0) was recorded in Writing, while above average performance (+) was recorded in Semantic Fluency. The results (“At Risk” Quotient >1.0) indicate that GA is strongly at risk of dyslexia (Table 1), highlighting his impairments not only in reading and writing skills but also in other cognitive processes [(meta)phonological awareness, memory, reasoning; Callens et al., 2012; Tops et al., 2013]. Considering the MMPI profile, moderate and low *T* scores in specific scales revealed a personality profile, with mild symptoms of anxiety and tension [two-point code type: 9-7/7-9, corresponding to Mania scale (Ma) and Psychasthenia scale (Pt)], although without symptoms of hypo-mania (or mania), or other forms of psychopathology (Table 1). This is a relatively rare code type that describes people with phobias, who tend to be self-centered and often immature. They may have periods of impulsive-reckless behavior that are a frequent cause of difficulties in interpersonal relationships. These results were combined with the patient’s history information and clinical characteristics, obtained in the context of a clinical interview.

**Table 1:** Scores of GA in the DAST and MMPI analyses.

The Dyslexia Adult Screening Test (DAST) GA’s profile			
Tasks assessed	Scores	“At Risk” Index scores	Interpreted scores
Rapid Naming	40	—	Very Strong Indicator
One Minute Reading	68	–	Strong Indicator
Postural Stability	2	-	Indicator
Phonemic Segmentation	5	—	Very Strong Indicator
Two Minute Spelling	22	–	Strong Indicator
Backwards Span	5	-	Indicator
Nonsense Passage	82	-	Indicator
Nonverbal Reasoning	3	–	Strong Indicator
One Minute Writing	30	0	Normal Band
Verbal Fluency	11	-	Indicator
Semantic Fluency	33	+	Above Average
**“At Risk” score**		**14**	
**“At Risk” Quotient (ARQ)**		**1,2**	**Strong “At Risk” Indicator**
**The Minnesota Multiphasic Personality Inventory (MMPI) GA’s profile**			
**Raw scores and *T*-scores of MMPI scales**			
		**Raw scores**	*T*-scores
**Validity Scales**
F (Infrequency)		8	53
L (Lie)		2	32
K (Correction)		9	40
**Clinical Scales**			
Hypochondriasis (Hs); Scale 1		12	46
Depression (D); Scale 2		18	41
Conversion Hysteria (Hy); Scale 3		15	43
Psychopathic Deviate (Pd); Scale 4		23	54
Masculinity-Femininity (Mf); Scale 5		26	51
Paranoia (Pa); Scale 6		11	54
**Psychasthenia (Pt); Scale 7**		29	**55**
Schizophrenia (Sc); Scale 8		26	50
**Hypomania (Ma); Scale 9**		25	**62**
Social Introversion (Si); Scale 0		28	50
**Case Profile**		**Code type: 9-7/7-9**	

*DAST: very severe difficulties (—) were recorded in the tasks of Rapid Naming and Phonemic Segmentation, while serious problems (–) were pointed out in One Minute Reading, Two Minute Spelling, and Nonverbal Reasoning, respectively. Noticeable problems (-) were observed in Postural Stability, Backwards Span, Nonsense Passage, and Verbal Fluency. The “At Risk” Quotient (>1.0) indicates that GA is strongly at risk of dyslexia. MMPI: profile of the scores on all of the MMPI scales. The patient obtained low *T*-scores in two validity (L and K) and two clinical scales (D and Hy), while moderate *T*-scores were recorded in one validity (F) and eight clinical scales (Hs, Pd, Mf, Pa, Pt, Sc, Ma, and Si). The two-point code type was determined by the highest two scales (shown in bold). *T*-scores were moderately elevated for Psychasthenia and Mania scale; however, none of the clinical scales showed elevations above 70*.

**Figure 1:**
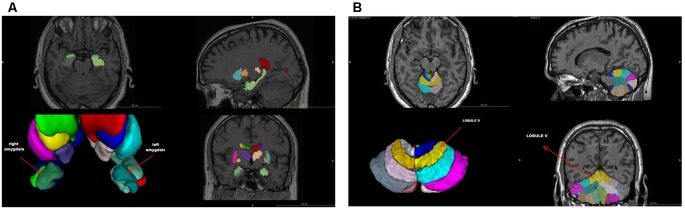
**(A)** Voxel-based morphometry (VMB) analysis of the cerebrum. **(B)** VBM lobular analysis of the cerebellum. The location of lobule V is shown (arrow).

The VBM analysis (Supplementary Table S1) revealed a smaller left amygdala volume compared to age- and sex-matched controls (GA: 0.03 vs. normal range 0.05–0.07), whereas the overall volume of the amygdala was close to the lowest normal value (GA: 0.08 vs. normal range 0.09–0.14). Indeed, the asymmetry index (calculated as the difference between right and left volumes divided by their mean) revealed a significant amygdala asymmetry (GA: +39.59 vs. normal range −16.41/+18.62; Figure 1A and Supplementary Table S1). No other significant differences were found (Supplementary Table S1). With regard to the cerebellum, the VBM lobular analysis showed that each lobule V was increased compared to controls (total; GA: 0.62 vs. normal range 0.21–0.42, right; GA: 0.32 vs. normal range 0.10–0.21, and left; GA: 0.30 vs. normal range 0.10–0.21; Figure 1B and Supplementary File S2). There was also an asymmetry in the volume of lobule Crus II, with the left one being smaller than the right (GA: +22.03 vs. normal range −22.45/+17.66; Supplementary Figure S1 and Supplementary File S2). The left lobule VIIIB was increased and close to the upper limit compared to age- and sex-matched controls (GA: 0.39 vs. normal range 0.19–0.39), while there was an asymmetry between the right and left lobule VIIIB, with the right one being smaller than the left in this case (GA: −36.48 vs. normal range −25.39/+30.73). Both lobules X were decreased (total; GA: 0.08 vs. normal range 0.33–0.70, right; GA: 0.04 vs. normal range 0.16–0.34, and left; GA: 0.04 vs. normal range 0.17–0.35; Supplementary File S2).

The VBM findings reveal anomalous anatomical asymmetries, which support the argument that the cerebellum is one of the main regions associated with dyslexia with consistent differences between critical groups (Eckert, 2004; Stoodley and Stein, 2011; Vandermosten et al., 2016). More importantly, the results from the lobular analysis and the DAST are consistent with the findings of Stanberry et al. (2006), namely, that the asymmetries of lobules V, Crus II, VIIB, and X are involved in difficulties of postural stability, rapid naming, rapid reading, phonemic segmentation, and memory. They are also in line with other studies showing that working memory and fluid intelligence are associated with the dorsolateral prefrontal and anterior cingulate cortex, and that phonological processing, implicit learning, and rapid automatized naming are related to cerebellar asymmetries (Molinari et al., 2008; Stoodley and Stein, 2011, 2013).

Interestingly, although GA was diagnosed with dyslexia quite early, he achieved advanced degrees, indicating that the recorded brain asymmetries do not merely represent differences in reading experience, but rather they may actually contribute with a causative role in dyslexia (Vandermosten et al., 2016). Worthy of mention is that the emotional and/or behavioral characteristics of GA’s profile are not classified in the clinical spectrum, indicating that: (i) the cerebellum asymmetries may be specific to his learning difficulties (Stoodley and Stein, 2011); and (ii) the amygdala asymmetries might reflect alterations in the expression of neuroplasticity genes, which could be triggered by the frustration that the persistent learning difficulties evoke as it has been previously suggested (Blair, 2010), affecting his behavior and stimulus reinforcement learning. However, it would have been certainly interesting to have access to information concerning earlier life periods of the subject examined, as to the form and degree of his dyslexia, because the data presented here only refer to adult findings (Shaywitz et al., 2002). Therefore, whenever possible, a temporal approach should be employed to correlate the critical developmental stages with the natural history of these patients.

**Figure 2:**
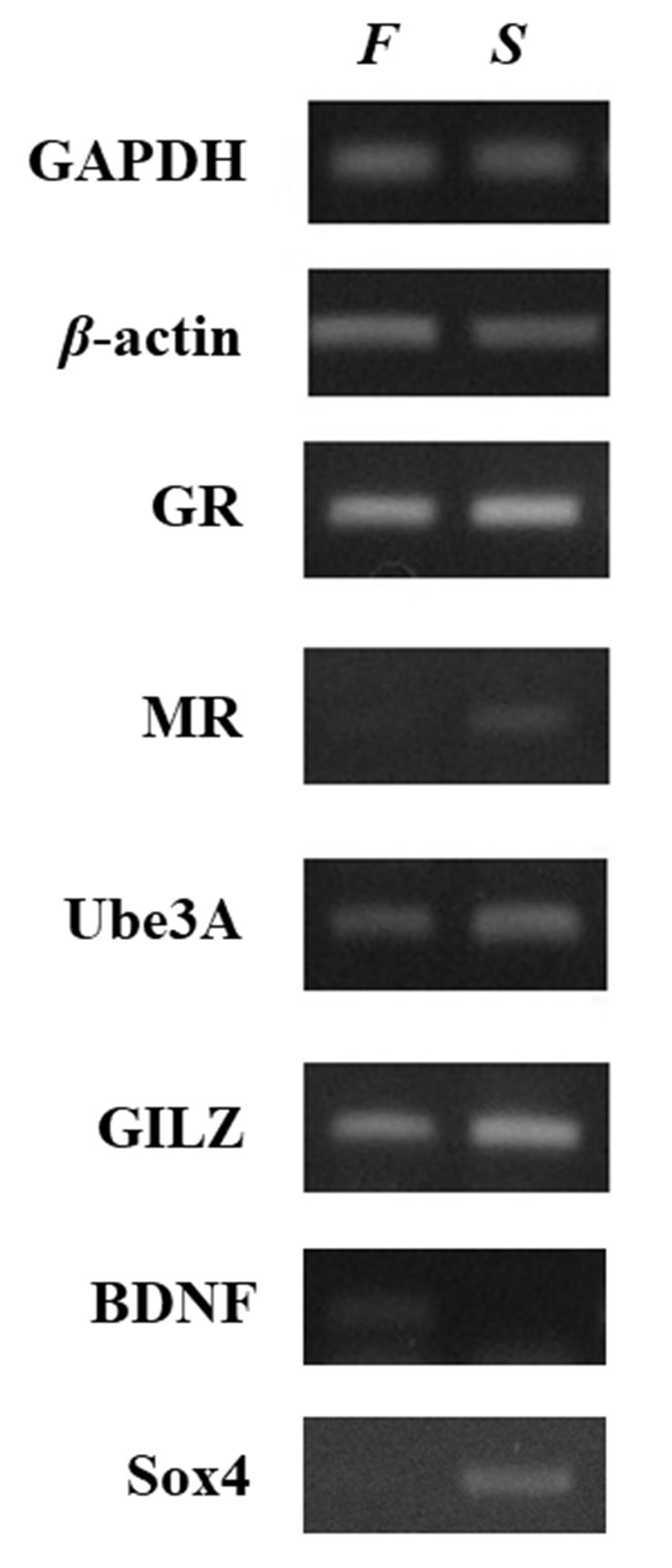
Relative expression of selected neuroplasticity and stress-related genes between father and son. Gene expression was compared in whole blood RNA samples collected from the patient and his son. Relative gene expression levels were determined by semiquantitative reverse transcriptase-polymerase chain reaction (RT-PCR). The levels of *GAPDH* and *β-actin* were used as controls for cDNA normalization. Photographs provide one representative example of at least three different experiments that yielded consistent results. The mRNA levels of the genes encoding *MR*, *Ube3A*, *GILZ*, and *Sox4* were lower in the sample from the father (*F*) compared to the son (*S*). In contrast, the expression of *BDNF* was significantly higher in the father whereas glucocorticoid receptor (GR) showed no difference.

The investigation of the gene expression profile was performed by reverse transcriptase-polymerase chain reaction (RT-PCR) using self-designed specific primers (Supplementary Table S2). This analysis (Figure 2) showed that the mRNA levels of the genes tested differed remarkably between the two samples, with the exception of *GR*, which showed no difference between the patient and his healthy child. The mRNA levels of *MR*, *UBE3A*, *GILZ*, and *Sox4* genes were lower in the father than in the son. In contrast, the expression of *BDNF* was significantly higher in the father. Several studies have recognized the role of epigenetic mechanisms in altering the expression of genes involved in HPA axis function and neuroplasticity after stressful conditions and experiences. Although the data have to be extended to include the analysis of more cases, the differences in the expression profiles of the genes observed here could reflect long-term adaptation and adjustments to changes in the environment. In other words, the father may have acquired epigenetic alterations in critical genes for brain function. One of the most remarkable differences was the very low expression of MR in the blood of the patient although the GR levels were similar. As MR and GR activities oppose each other, the ratio of MR to GR is considered as a marker for stress resilience and vulnerability (Almeida et al., 2000). Provided that this is reflected in relevant brain regions, it may denote an imbalance between GR- and MR-mediated actions in the limbic system, which may lead to inadequate response to stress (de Kloet, 2013). It has been suggested that, during stress, MR provides a negative feedback signal, and low MR functionality may predispose individuals to increased stress susceptibility for psychiatric disorders (Harris et al., 2013). In addition, reduced MR expression in the limbic system may lead to a less favorable strategy to respond properly to a novel, stressful situation (ter Horst et al., 2012). Thus, decreased MR levels may affect stress-related learning and modify the cognitive appraisal of stressful situations (ter Heegde et al., 2015). Another gene, the mRNA levels of which were significantly lower in the father compared to the son, was that of *Sox4*, which may reflect impaired neurogenesis, and therefore insufficient supply with new neurons that could become integrated into pre-existing neuronal networks. The levels of BDNF mRNA were notably higher in the patient, possibly indicating chronic compensatory mechanisms to cope with stressful events (Autry and Monteggia, 2012; Zaletel et al., 2017).

The analysis of peripheral blood is emerging as a pertinent route for studying relevant gene expression changes in brain disease. For example, chromatin extracted from peripheral blood carries epigenetic marks that reflect individual life experiences. Such epigenetic biomarkers have been associated with various brain disorders, including schizophrenia (Gavin and Sharma, 2009), depression, aggressive behavior, or post-traumatic stress disorder (Rusiecki et al., 2012). Moreover, *GILZ* mRNA levels in peripheral blood mononuclear cells have been correlated with hippocampal volumes in patients with depression (Frodl et al., 2012). In addition, the methylation of the GR promoter in blood leukocytes has been associated with the history of various childhood adversities (McGowan et al., 2009; Tyrka et al., 2012), while acute psychosocial stress has been shown to alter the DNA methylation status of the *BDNF* gene in peripheral blood cells (Unternaehrer et al., 2012).

## Concluding Remarks

**Figure 3:**
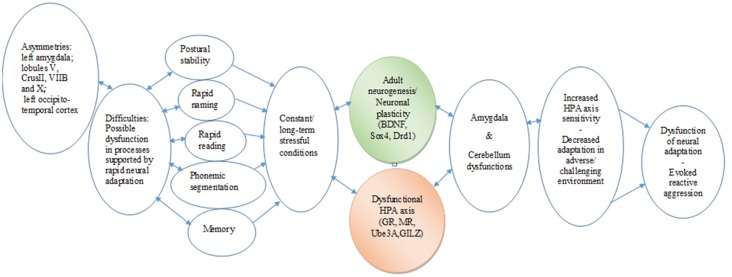
Chain link of brain asymmetries, specific learning disabilities, neurogenesis, hypothalamic–pituitary–adrenal (HPA) axis, and psycho-emotional disorders. The proposed translational mechanism highlights the complex roles that specific brain and genetic asymmetries in constant interaction with the HPA axis may play in the expression and the management of learning disabilities. It also indicates the longitudinal potential consequences in cognitive and emotional development and in the behavioral adaptation of the individual from early childhood to adulthood (Hoeft et al., 2011). In essence, this chain link underscores the core roles of multifaceted associations between neurophysiological and epigenetic development, as well as the adaptation of cognitive and learning mechanisms.

The data analyses on GA point to a dyslexic profile or endophenotype, characterized by altered stress response, MRI findings (asymmetries in the amygdala and specific cerebellar regions), and difficulties in coping with strong emotional and behavioral states. The case findings can be taken to indicate that the cerebellum asymmetries may negatively affect the skills of rapid reading and writing, phonological segmentation and sensorimotor functionality, possibly resulting in a blunted stress response. The asymmetries of the amygdala could indicate an impaired regulation system of the response to threatening stimuli (reading difficulties), which may result in reduced GR-mediated negative feedback on the HPA axis and allow reactive aggression. Given that the long-standing reading difficulties mirror threatening of frustrating conditions, they are considered as stress stimuli; as a result, the limited amygdala activity unable to manipulate negative emotions, supported by a corticosteroid receptor imbalance, might compromise adaptation and activate reactive aggression. In addition, a potential interplay between the dysfunction of particular lobules and amygdala should be taken into account, considering the dynamic changes in gene expression that may occur in the amygdala under threatening conditions, such as the learning difficulties. The significant differences in the expression profiles of the HPA axis and the neuroplasticity genes tested here may reflect long-term adjustments of transcriptional programs to the “threatening” learning environment (Figure 3). Thus, stress might be an important environmental factor that could act in concordance with genetic mutations or alone (epigenetically), resulting in DD endophenotypes and a variation of clinical phenotypes.

Thus, we propose that the present single case report suggests that, for a more integrated diagnosis and effective treatment, stress-related phenotypes should be carefully considered; in this direction, one should also bear in mind that epigenetic changes induced by environmental factors are dynamic and could be reversed with appropriate intervention processes. This interpretation is in line with current conceptualizations that, in the analysis of complex behaviors (for example, learning disabilities), different components such as cognitive processes, neural systems, and genetic and epigenetic factors should be co-estimated. While the in-depth description of a single case may be highly suggestive, future research involving a sizeable sample of informative individuals will be definitely required in order to reach firm conclusions on this interpretation.

## Data Availability Statement

All datasets analyzed for this study are included in the manuscript/Supplementary files.

## Ethics Statement

The patient and his son provided us with written informed consent in accordance with the Declaration of Helsinki. They also gave their consent for the analysis, processing, and publication of the data. Since all described interventions were part of clinical practice, we did not consult the ethics committee for this study.

## Author Contributions

VZ, MS and TM conceived and designed the experiments and contributed reagents, materials and analysis tools. VZ, A-MV, MD, ZP, DT, KP, GA, HB, VS, MS and TM performed the experiments. VZ, GA, HB, VS, PZ, GC, MS and TM analyzed the data. VZ, A-MV, MD, ZP, DT, GA, HB, VS, PZ, GC, MS and TM wrote the article.

## Conflict of Interest

The authors declare that the research was conducted in the absence of any commercial or financial relationships that could be construed as a potential conflict of interest.

## Supplementary Material

SUPPLEMENTARY FILE S1 Materials and Methods.

SUPPLEMENTARY FILE S2 Volumetry report: the raw data are shown, including a screenshot of the results.

FIGURE S1 VBM lobular analysis of the cerebellum: the location of lobule Crus II is shown (arrow).

TABLE S1 Volumetric brain analysis.

TABLE S2 Primer sequences used for reverse transcription-PCR.
