# Supplementary material for: Linking Early Life Hypothalamic–Pituitary–Adrenal Axis Functioning, Brain Asymmetries, and Personality Traits in Dyslexia: An Informative Case Study
Source: Front Hum Neurosci. 2019 Oct 1;13:327. doi: 10.3389/fnhum.2019.00327 (PMC6779713; doi:10.3389/fnhum.2019.00327)
Supplement: SUPPLEMENTARY FILE S1 — Materials and Methods. [file Data_Sheet_1.pdf]

MATERIALS AND METHODS

The Dyslexia Adult Screening Test (DAST)

The DAST is designed to provide core information about the classic dyslexic symptoms and signs that an adult could present with. The test provides a profile of adults' strengths and weaknesses in reading, writing, and spelling skills, which could be provided as an "at risk" index of dyslexia. It contains 11 sub-tests recording the achievement in the following particular areas: Rapid Naming; One Minute Reading; Postural Stability; Phonemic Segment; Two Minute Spelling; Backwards Span; Nonsense Passage; Nonverbal Reasoning; One Minute Writing; Verbal Fluency; Semantic Fluency. The scoring in each sub-test of the DAST indicates the degree of "risk" for occurrence of dyslexia, as follows: [---]:"very strong indicator", [--]: "strong indicator", and [-]: "indicator".

The Minnesota Multiphasic Personality Inventory (MMPI)

The Minnesota Multiphasic Personality Inventory (MMPI) is a psychologic test that assesses personality traits and psychopathology (Hathaway and McKinley, 1951). The MMPI consists of 566 items (all true or false format) and takes approximately 60 to 90 minutes to complete. Ten clinical scales are used in assessment: Hypochondriasis (Hs), Depression (D), Hysteria (Hy), Psychopathic deviation (Pd), Masculinity - Femininity (Mf), Paranoia (Pa), Psychasthenia (Ps), Schizophrenia (Sc), Mania (Ma) and Social Introversion (Si). In addition, there are three validity scales (L, F and K) to assess the response style (i.e., cooperative). The Lie Scale (L) reflects how the individual took the test and if he/she attempted to give an overly good view of himself / herself (faking good). The Rare Answer Scale (F) measures the tendency to give statistically rare responses (faking bad) and the "Correction" Scale (K) was designed to measure "normality" or "defensiveness". In general, high K reflects defensiveness, social desirability, formality and an effort to hide something. For both, clinical and validity scales, raw scores are transformed into T scores. A T score ≥ 70 is considered as pathological, a T score between 50 and 65 as moderate, and a T score <45 as low. The profile interpretation was based on a two-point code type, which is defined by the two highest-scoring clinical scales. A code type is construed as a single, wider ranged elevation with more clinical significance than just interpreting each scale individually (Groth-Marnat, 2003).

Blood Collection and Analysis of Gene Expression

Blood from the patient and his son was collected in the morning, and stored at −20 °C. RNA was isolated from 1 ml peripheral blood using a standard protocol kit (Qiagen, #52304) according to the manufacturers' recommendations. A total volume of 30μl eluted RNA was stored at -80°C for next step procedures. The quantity and quality of the RNA samples were measured using a spectrophotometer (NanoDrop) and ethidium bromide (EB) visualization of intact 18S and 28S RNA bands after agarose gel electrophoresis.

Reverse transcription (RT) was carried out using 0.5μg of total RNA. The standard protocol for "Perfect Real-Time" was performed according to the Reverse Transcriptase kit (TAKARA, #RR047A) and the cDNAs were stored at -20°C until further analysis. Relative transcript levels were assessed by semi-quantitative RT-PCR (Polymerase Chain Reaction) analysis. Gene-specific primers for the genes *NR3C1*, *NR3C2*, *UBE3A*, *GILZ*, *POMC* and *BDNF*, as well as of the two housekeeping genes *β-actin* and *GAPDH* were designed according to the corresponding NCBI GenBank sequences, and are presented in **Supplementary Table 2**. All primers were synthesized by the Microchemistry Facility of the Institute of Molecular Biology and Biotechnology (Heraklion, Greece).

PCR reactions were carried out in 50 μl mixtures containing 1/20th volume of the cDNA preparation, 10 μl of 5x standard PCR buffer, 1 μl of 10 mM dNTPs, 1 μl of 25 μM of each primer and 2.5 units Taq polymerase (Minotech Biotechnology, IMBB, Greece). The cycling program was: step 1: 94°C

for 3 min, followed by 35-40 cycles of step 2: 94°C for 30 sec, 59°C for 30 sec, 72°C for 1 min, extension step 3: 72°C for 7 min and final step 4: 4°C until the end of the program. The PCR products were loaded on 1.6% agarose gels stained with EtBr and electrophoresed in 80V for almost 40 minutes. A DNA ladder 100bp was used as a marker to determine the PCR amplicon sizes (*NR3C1*: 201bp, *NR3C2*: 192bp, *UBE3A*: 161bp, *GILZ*: 199bp, *BDNF*: 131bp, *GAPDH*: 200bp, *β-actin*: 141bp).

Magnetic Resonance Imaging and 3-Dimensional Surface Rendering Techniques
All images were acquired in a 1.5 Tesla MRI scanner using a three-dimensional spoiled gradient recall acquisition in the steady state (time to echo, 4.6 milliseconds; time to repeat, 25 milliseconds; flip angle, 30 degrees; repetition, 1; field of view, 250 cm^2). Contiguous axial slices, 1 mm thickness (150 per brain), were obtained. The images were collected in a 256x256 acquisition matrix and resulted in an effective voxel resolution of approximately 1x1x1 mm.

Computerized Image Analysis
All images were transferred to a computer, and a voxel based morphometry (VBM) analysis was performed using volBrain (Manjón and Coupé, 2016), an MRI brain volumetry system that provides volume information of some macroscopic areas, such as brain hemispheres, cerebellum and brainstem. Finally, automatic subcortical structure segmentation was performed and related volumes and label maps were computed. In this analysis (**Supplementary Table 1** and **Supplementary File 2**) all volumes (total, left and right hemispheres) are presented in absolute values as well as in relative values (%) measured in relation to the intracranial volume (ICV) and compared to the normal reference range. The expected lower and upper bounds of the normalized volumes in function of sex and age for each measure are shown in brackets. The Asymmetry Index is calculated as the difference between right and left volumes divided by their mean (in percent). The methodology followed for the quantitative validation of the asymmetries, as measured by the VBM analysis is available through the volBrain online web interface (http://volbrain.upv.es). The method is public and freely accessible to the scientific community.
