# Supplementary material for: Linking Early Life Hypothalamic–Pituitary–Adrenal Axis Functioning, Brain Asymmetries, and Personality Traits in Dyslexia: An Informative Case Study
Source: Front Hum Neurosci. 2019 Oct 1;13:327. doi: 10.3389/fnhum.2019.00327 (PMC6779713; doi:10.3389/fnhum.2019.00327)
Supplement: SUPPLEMENTARY FILE S2 — Volumetry report: the raw data are shown, including a screenshot of the results. [file Data_Sheet_2.pdf]

CERES Volumetry Report. version 1.0 release 12-04-2016

Patient ID	Sex	Age	Report Date
job8313	Male	56	14-Apr-2016

Image Information	
Orientation	radiological
Scale factor	0.77
SNR	23.28
Total intracranial volume (cm^3)	1400.67

Volumes	Total (cm^3/%)	Right (cm^3/%)	Left (cm^3/%)	Asym.(%)
Cerebellum	123.61 (8.8250)	61.91 (4.4198)	61.70 (4.4053)	0.3297
	[7.5568, 10.2191]	[3.7565, 5.0920]	[3.7911, 5.1362]	[-4.3444, 2.5936]
Lobule I-II	0.17 (0.0118)	0.07 (0.0053)	0.09 (0.0065)	-21.3953
	[-0.0023, 0.0259]	[-0.0030, 0.0156]	[0.0002, 0.0107]	[-51.8747, 62.8885]
Lobule III	1.68 (0.1199)	0.80 (0.0573)	0.88 (0.0626)	-8.8787
	[0.0682, 0.1471]	[0.0319, 0.0738]	[0.0331, 0.0764]	[-31.8751, 25.3507]
Lobule IV	4.06 (0.2901)	2.02 (0.1445)	2.04 (0.1455)	-0.6813
	[0.2189, 0.4226]	[0.1097, 0.2167]	[0.1014, 0.2137]	[-20.5661, 27.6084]
Lobule V	8.74 (0.6242)	4.50 (0.3212)	4.24 (0.3030)	5.8570
	[0.2189, 0.4226]	[0.1097, 0.2167]	[0.1014, 0.2137]	[-20.5661, 27.6084]
Lobule VI	14.17 (1.0114)	6.84 (0.4882)	7.33 (0.5231)	-6.9040
	[0.9112, 1.5182]	[0.4493, 0.7588]	[0.4482, 0.7731]	[-16.0585, 14.1819]
Lobule Crus I	28.37 (2.0257)	14.30 (1.0209)	14.07 (1.0048)	1.5826
	[1.3328, 2.2622]	[0.6396, 1.1271]	[0.6719, 1.1565]	[-19.0268, 12.0804]
Lobule Crus II	14.71 (1.0502)	8.17 (0.5830)	6.54 (0.4673)	22.0364
	[0.7886, 1.4683]	[0.3832, 0.7304]	[0.3863, 0.7570]	[-22.4576, 17.6638]
Lobule VIIB	7.88 (0.5627)	4.32 (0.3083)	3.56 (0.2544)	19.1786
	[0.4507, 0.8272]	[0.2137, 0.4223]	[0.2175, 0.4244]	[-28.6581, 26.7682]
Lobule VIIIA	11.09 (0.7921)	5.45 (0.3889)	5.65 (0.4033)	-3.6313
	[0.6270, 1.0234]	[0.3051, 0.5191]	[0.2974, 0.5287]	[-23.8066, 24.0274]
Lobule VIIIB	9.30 (0.6638)	3.80 (0.2713)	5.50 (0.3924)	-36.4869
	[0.4240, 0.7684]	[0.2044, 0.3996]	[0.1982, 0.3902]	[-25.3966, 30.7348]
Lobule IX	6.64 (0.4738)	3.22 (0.2300)	3.42 (0.2438)	-5.8387
	[0.3371, 0.7044]	[0.1628, 0.3477]	[0.1718, 0.3592]	[-15.8072, 7.6460]
Lobule X	1.18 (0.0844)	0.59 (0.0419)	0.60 (0.0425)	-1.5605
	[0.3371, 0.7044]	[0.1628, 0.3477]	[0.1718, 0.3592]	[-15.8072, 7.6460]

**All the volumes are presented in absolute value (measured in cm^3) and in relative value (measured in relation to the ICV).*

**The Asymmetry Index is calculated as the difference between right and left volumes divided by their mean (in percent).*

**Cortical thickness is given in absolute value (mm) and also normalized in relation to the cube root of the intracranial volume (adimensional).*

**Result images located in the MNI space (neurological orientation).*

**Grey matter vol.**	**Total** (cm^3/%)	**Right** (cm^3/%)	**Left** (cm^3/%)	**Asym.**(%)
Cerebellum	89.32 (6.3768)	44.80 (3.1987)	44.52 (3.1781)	0.6439
	[5.2813, 7.8043]	[2.6287, 3.8817]	[2.6433, 3.9318]	[-5.5657, 3.6764]
Lobule I-II	0.11 (0.0079)	0.05 (0.0033)	0.06 (0.0046)	-43.3529
	[-0.0035, 0.0192]	[-0.3395, 0.0117]	[-0.0004, 0.0079]	[-90.2092, 81.4354]
Lobule III	1.41 (0.1006)	0.66 (0.0474)	0.75 (0.0532)	-15.0505
	[0.0516, 0.1227]	[2.4521, 0.0622]	[0.0244, 0.0632]	[-38.0064, 36.2989]
Lobule IV	3.67 (0.2620)	1.86 (0.1328)	1.81 (0.1292)	3.6513
	[0.1869, 0.3765]	[9.3687, 0.1953]	[0.0862, 0.1881]	[-24.4861, 37.3067]
Lobule V	7.75 (0.5532)	4.02 (0.2870)	3.73 (0.2661)	9.8347
	[0.1869, 0.3765]	[9.3687, 0.1953]	[0.0862, 0.1881]	[-24.4861, 37.3067]
Lobule VI	12.58 (0.8983)	6.16 (0.4397)	6.42 (0.4586)	-5.4681
	[0.7890, 1.3715]	[39.2706, 0.6896]	[0.3842, 0.6940]	[-18.6403, 19.8643]
Lobule Crus I	20.44 (1.4590)	10.28 (0.7343)	10.15 (0.7248)	1.6931
	[0.9955, 1.8081]	[48.2569, 0.9003]	[0.4942, 0.9265]	[-24.9316, 18.4447]
Lobule Crus II	10.38 (0.7407)	5.65 (0.4031)	4.73 (0.3376)	23.0162
	[0.6017, 1.1456]	[28.9278, 0.5674]	[0.2954, 0.5953]	[-31.1551, 22.0556]
Lobule VIIB	6.62 (0.4727)	3.62 (0.2586)	3.00 (0.2142)	24.4049
	[0.3727, 0.6856]	[17.3093, 0.3445]	[0.1832, 0.3575]	[-39.6971, 28.6010]
Lobule VIIIA	9.75 (0.6961)	4.88 (0.3487)	4.87 (0.3474)	0.4923
	[0.5104, 0.8786]	[24.9211, 0.4433]	[0.2419, 0.4546]	[-29.7371, 29.4163]
Lobule VIIIB	8.17 (0.5832)	3.35 (0.2391)	4.82 (0.3441)	-46.8133
	[0.3514, 0.6622]	[17.0125, 0.3405]	[0.1652, 0.3377]	[-31.7603, 35.7068]
Lobule IX	5.76 (0.4112)	2.77 (0.1979)	2.99 (0.2133)	-9.7574
	[0.2479, 0.5937]	[11.8728, 0.2892]	[0.1270, 0.3066]	[-23.7011, 8.2414]
Lobule X	1.15 (0.0822)	0.58 (0.0411)	0.58 (0.0411)	-0.1738
	[0.2479, 0.5937]	[11.8728, 0.2892]	[0.1270, 0.3066]	[-23.7011, 8.2414]

**All the volumes are presented in absolute value (measured in cm^3) and in relative value (measured in relation to the ICV).*

**The Asymmetry Index is calculated as the difference between right and left volumes divided by their mean (in percent).*

**Cortical thickness is given in absolute value (mm) and also normalized in relation to the cube root of the intracranial volume (adimensional).*

**Result images located in the MNI space (neurological orientation).*

**Cortical thickness**	**Mean** ($mm/norm.$)	**Right** ($mm/norm.$)	**Left** ($mm/norm.$)	**Asym.**(%)
Cerebellum	4.39 (3.924)	4.37 (3.906)	4.41 (3.943)	0.9551
	[3.459, 4.043]	[3.418, 4.051]	[3.468, 4.066]	[-4.2639, 6.0397]
Lobule I-II	2.71 (2.422)	2.80 (2.504)	2.64 (2.359)	-6.0125
	[0.930, 3.363]	[0.964, 3.404]	[0.876, 3.326]	[-22.8700, 15.2214]
Lobule III	4.30 (3.847)	4.25 (3.802)	4.35 (3.886)	2.1984
	[2.672, 4.060]	[2.725, 4.141]	[2.566, 4.022]	[-15.0263, 6.5772]
Lobule IV	4.93 (4.406)	4.97 (4.445)	4.88 (4.366)	-1.7932
	[3.997, 4.593]	[4.029, 4.630]	[3.923, 4.590]	[-6.7078, 3.2760]
Lobule V	4.94 (4.415)	4.96 (4.430)	4.92 (4.400)	-0.6627
	[3.997, 4.593]	[4.029, 4.630]	[3.923, 4.590]	[-6.7078, 3.2760]
Lobule VI	4.87 (4.351)	4.83 (4.317)	4.90 (4.383)	1.5034
	[3.889, 4.558]	[3.883, 4.599]	[3.857, 4.553]	[-6.1518, 4.4138]
Lobule Crus I	3.92 (3.500)	3.94 (3.521)	3.89 (3.479)	-1.2146
	[2.901, 3.935]	[2.851, 3.945]	[2.858, 4.003]	[-11.7450, 13.5573]
Lobule Crus II	3.25 (2.904)	3.15 (2.811)	3.37 (3.016)	7.0566
	[2.430, 3.791]	[2.183, 3.754]	[2.553, 3.927]	[-9.9144, 27.9915]
Lobule VIIB	4.58 (4.093)	4.60 (4.112)	4.55 (4.070)	-1.0322
	[3.240, 4.321]	[2.975, 4.369]	[3.406, 4.347]	[-7.8078, 19.1444]
Lobule VIIIA	4.81 (4.296)	4.88 (4.360)	4.73 (4.230)	-3.0259
	[3.555, 4.412]	[3.484, 4.484]	[3.544, 4.415]	[-9.4583, 9.2834]
Lobule VIIIB	4.95 (4.424)	4.93 (4.409)	4.96 (4.433)	0.5416
	[3.665, 4.542]	[3.643, 4.625]	[3.542, 4.601]	[-14.3045, 11.2217]
Lobule IX	4.59 (4.101)	4.56 (4.078)	4.61 (4.122)	1.0667
	[2.553, 4.113]	[2.435, 4.054]	[2.601, 4.225]	[-7.8679, 18.2528]
Lobule X	3.47 (3.104)	3.71 (3.312)	3.22 (2.882)	-13.8273
	[2.553, 4.113]	[2.435, 4.054]	[2.601, 4.225]	[-7.8679, 18.2528]

**All the volumes are presented in absolute value (measured in cm^3) and in relative value (measured in relation to the ICV).*

**The Asymmetry Index is calculated as the difference between right and left volumes divided by their mean (in percent).*

**Cortical thickness is given in absolute value (mm) and also normalized in relation to the cube root of the intracranial volume (adimensional).*

**Result images located in the MNI space (neurological orientation).*

Intracranial cavity extraction

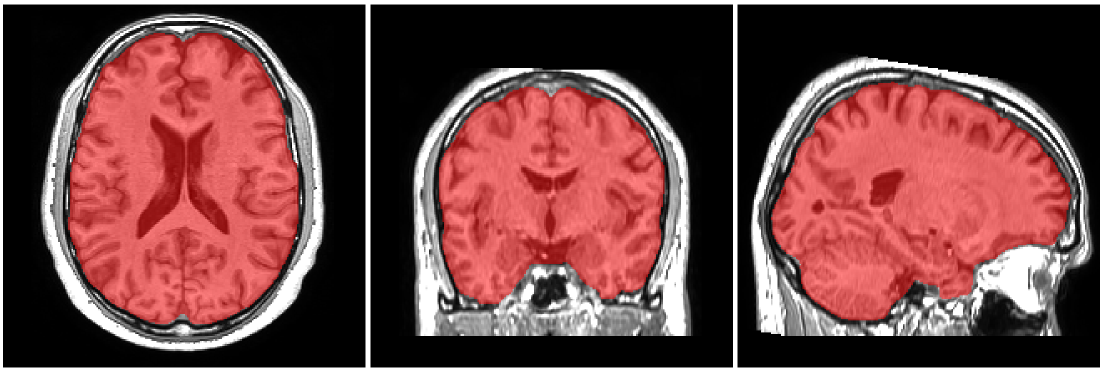

Lobules segmentation

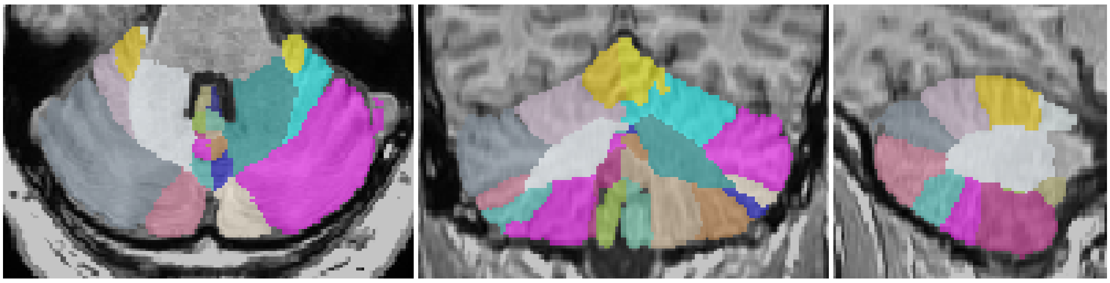

Tissue classification

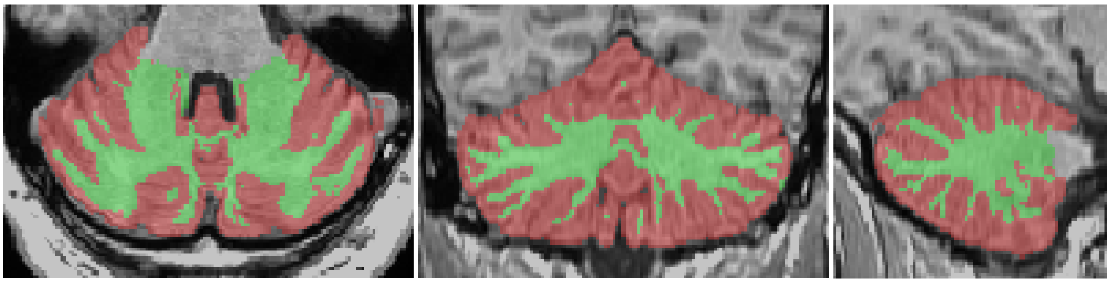

Cortical thickness

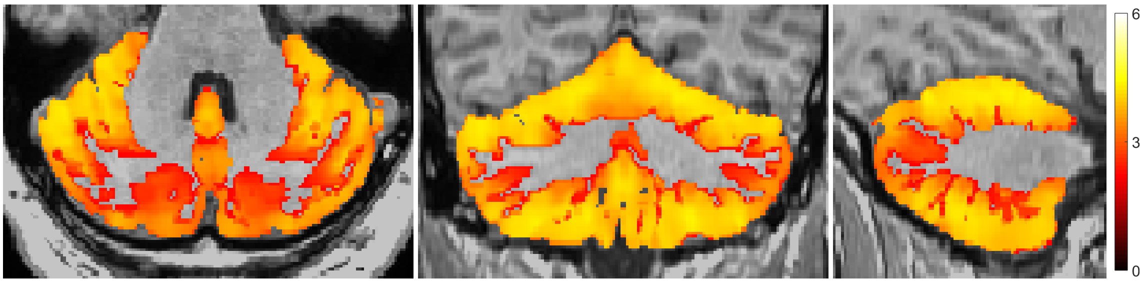

All the volumes are presented in absolute value (measured in cm^3) and in relative value (measured in relation to the ICV).

The Asymmetry Index is calculated as the difference between right and left volumes divided by their mean (in percent).

Cortical thickness is given in absolute value (mm) and also normalized in relation to the cube root of the intracranial volume (adimensional).

Result images located in the MNI space (neurological orientation).
