# Supplementary figures and images for: Linking Early Life Hypothalamic–Pituitary–Adrenal Axis Functioning, Brain Asymmetries, and Personality Traits in Dyslexia: An Informative Case Study
Source: Front Hum Neurosci. 2019 Oct 1;13:327. doi: 10.3389/fnhum.2019.00327 (PMC6779713; doi:10.3389/fnhum.2019.00327)

SUPPLEMENTARY FIGURE 1

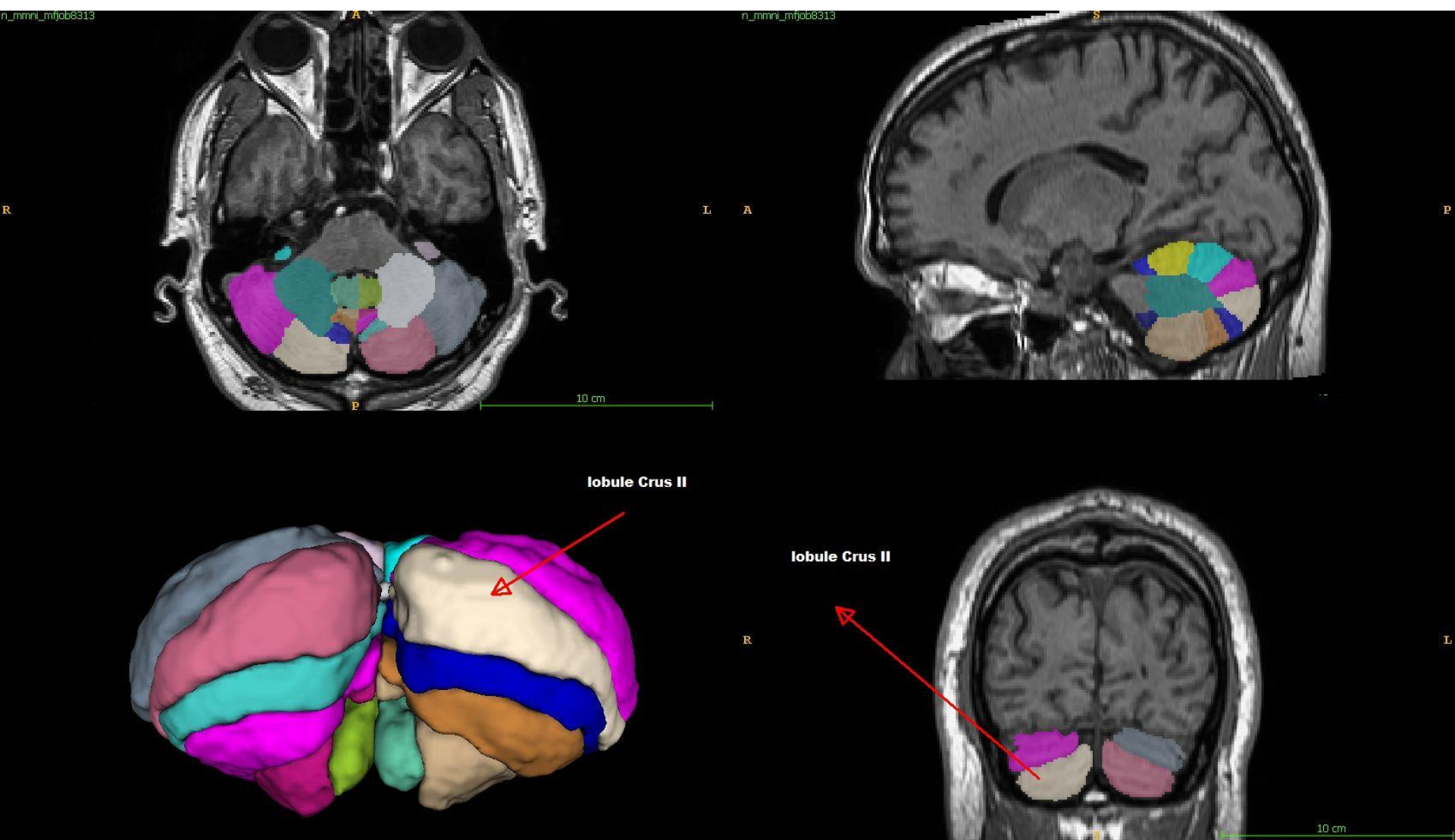

Supplement: FIGURE S1 — VBM lobular analysis of the cerebellum: the location of lobule Crus II is shown (arrow). [file Image_1.pdf]
