# Supplementary material for: Linking Early Life Hypothalamic–Pituitary–Adrenal Axis Functioning, Brain Asymmetries, and Personality Traits in Dyslexia: An Informative Case Study
Source: Front Hum Neurosci. 2019 Oct 1;13:327. doi: 10.3389/fnhum.2019.00327 (PMC6779713; doi:10.3389/fnhum.2019.00327)
Supplement: TABLE S1 — Volumetric brain analysis. [file Table_1.pdf]

SUPPLEMENTARY TABLE 1 Volumetric brain analysis.

Tissue type	Volume (cm^3/%)		Image information	
White Matter (WM)	554.66 (39.52%)	[31.95- 44.17]	Orientation	radiological
Grey Matter (GM)	683.35 (48.69%)	[40.96-52.24]	Scale factor	0.77
Cerebro Spinal Fluid (CSF)	165.53 (11.79%)	[10.03- 20.64]	SNR	39.76
Brain (WM + GM)	1238.01 (88.21%)	[79.36- 89.97]		
Intracranial Cavity (IC)	1403.55 (100.00%)			

Structure

Cerebrum	Total (cm^3/%)		Right (cm^3/%)		Left (cm^3/%)		Asym.(%)
	1081.15 (77.03%)		539.46 (38.44%)		541.69 (38.59%)		-0.4131
	[68.52 - 78.61]		[34.27 - 39.46]		[34.21 - 39.19]		[-1.24 - 2.15]
	GM	WM	GM	WM	GM	WM	
	583.73 (41.59%)	497.43 (35.44%)	292.36 (20.83%)	247.10 (17.61%)	291.37 (20.76%)	250.33 (17.84%)	
	[34.55- 44.06]	[28.87,-39.65]	[17.28- 22.06]	[14.44- 19.95]	[17.25,-22.01]	[14.41- 19.72]	

Cerebellum	Total (cm^3/%)		Right (cm^3/%)		Left (cm^3/%)		Asym.(%)
	133.44 (9.51%)		66.23 (4.72%)		67.21 (4.79%)		-1.4680
	[8.11- 10.66]		[4.02- 5.35]		[4.06- 5.33]		[-5.17- 4.62]
	GM	WM	GM	WM	GM	WM	
	93.25 (6.64%)	40.19 (2.86%)	44.88 (3.20%)	21.35 (1.52%)	48.37 (3.45%)	18.84 (1.34%)	
	[5.64- 8.30]	[1.56- 3.26]	[2.76- 4.13]	[0.79- 1.69]	[2.87- 4.19]	[0.76-1.58]	

Brainstem	Total (cm^3/%)
	23.50 (1.67%) [1.47 - 1.97]

Structure	Total (cm^3/%)	Right (cm^3/%)	Left (cm^3/%)	Asymmetry (%)
Lateral ventricles	16.94 (1.21%) [0.01-2.44]	6.31 (0.45%) [0.00-1.23]	10.63 (0.76%) [0.01- 1.26]	-50.9779 [-68.9071-54.97]
Caudate	7.61 (0.54%) [0.37-0.56]	3.94 (0.28%) [0.19- 0.28]	3.67 (0.26%) [0.18-0.28]	6.9871 [-5.3096-9.65]
Putamen	7.34 (0.52%) [0.44-0.65]	3.71 (0.26%) [0.22-0.32]	3.63 (0.26%) [0.22-0.33]	2.2213 [-7.5205- 4.99]
Thalamus	10.16 (0.72%) [0.64-0.85]	5.09 (0.36%) [0.32-0.42]	5.07 (0.36%) [0.32-0.43]	0.4994 [-8.8083-5.47]
Globus Pallidus	2.08 (0.15%) [0.13-0.20]	1.01 (0.07%) [0.06-0.10]	1.07 (0.08%) [0.06-0.10]	-6.5755 [-11.4260- 13.27]
Hippocampus	8.14 (0.58%) [0.46-0.65]	3.99 (0.28%) [0.23-0.33]	4.15 (0.30%) [0.23-0.32]	-3.9290 [-8.9401- 13.08]
Amygdala	**1.20 (0.08%)** [0.09-0.14]	0.72 (0.05%) [0.04-0.07]	**0.48 (0.03%)** [0.05- 0.07]	**39.5900** [-16.4169- 18.62]
Accumbens	0.60 (0.04%) [0.03- 0.06]	0.29 (0.02%) [0.01-0.03]	0.31 (0.02%) [0.01- 0.03]	-8.9400 [-40.4389-12.99]

* *Normalization according to normal matched control to sex and age. The significant differences are marked with red color.*

* *All the volumes are presented in absolute value (measured in* cm^3*) and in relative value (measured in relation to the ICV).*

* *The Asymmetry Index is calculated as the difference between right and left volumes divided by their mean (in percent).*

* *Expected lower and upper bounds (95%) of normalized volume in function of sex and age for each measure are provided (between brackets) for reference purpose.*
