# Supplementary material for: Linking Early Life Hypothalamic–Pituitary–Adrenal Axis Functioning, Brain Asymmetries, and Personality Traits in Dyslexia: An Informative Case Study
Source: Front Hum Neurosci. 2019 Oct 1;13:327. doi: 10.3389/fnhum.2019.00327 (PMC6779713; doi:10.3389/fnhum.2019.00327)
Supplement: TABLE S2 — Primer sequences used for reverse transcription-PCR. [file Table_2.pdf]

SUPPLEMENTARY TABLE 2 Primer sequences used for reverse transcription-PCR.

**Gene name**	**NCBI Reference Sequence**	**Sequence (5'- 3')**
NR3C1<br>*nuclear receptor subfamily 3 group C member 1*	NM_001204264.1	TGAAATGGGCAAAGGCGATACC<br>GGTCATACATGCAGGGTAGAGT
NR3C2<br>*nuclear receptor subfamily 3 group C member 2*	NM_000901.4	GTGTCCCAACAATTCTGGGCAG<br>GACTCCACCTTGGGCAGCT
UBE3A<br>*ubiquitin protein ligase E3A*	NM_130838.1	GGCTTTTCGGAGAGGTTTTCAT<br>CAGAGTCCCTGGTATAGCCACC
GILZ<br>*TSC22 domain family member 3 (TSC22D3)*	NM_198057.2	CAAGATTGAGCAGGCCATGGA<br>TGGCTCTTCAGGGCTCAGC
BDNF<br>*brain-derived neurotrophic factor*	NM_170735.5	TGAGTCTCCAGGACAGCAAAGC<br>GTGGACGTTTACTTCTTTCATGG
β-actin	NM_001101.3	TTGCTGACAGGATGCAGAAG<br>ACATCTGCTGGAAGGTGGAC
GAPDH	NM_002046.5	GTGGTCTCCTCTGACTTCAACA<br>ACCACCCTGTTGCTGTAGCC
